# Transition to Reusable Surgical Gowns at a Hospital System

**DOI:** 10.1001/jamanetworkopen.2023.30246

**Published:** 2023-08-22

**Authors:** Ava Yap, Kaiyi Wang, John Cornejo, Evan Chen, Logan Pierce, Elizabeth Wick, Seema Gandhi

**Affiliations:** 1 University of California, San Francisco

## Abstract

This quality improvement study investigates the feasibility of transitioning back to reusable surgical gowns at a US tertiary hospital system and projected corresponding cost savings and solid waste reduction.

## Introduction

The US health care system is tremendously wasteful, and 70% of waste comes from operating rooms.^1^ Waste production grew by 15% over 20 years as disposable surgical gowns replaced reusable cotton surgical gowns and more surgical equipment became single-use.^2^ Reusable surgical gowns are an environmentally friendly alternative and were a norm of the past.^3^ We examined the feasibility of transitioning back to reusable surgical gowns at a US tertiary hospital system and projected corresponding cost savings and solid waste reduction.

## Methods

The University of California, San Francisco, institutional review board deemed this quality improvement study exempt and informed consent was waived due to the anonymous nature of this study. We followed the SQUIRE 2.0 reporting guideline.^4^

This study was conducted at a tertiary hospital system in California that supplied only disposable surgical gowns from October 2021 to April 2022. Polyester reusable surgical gowns with Association of Advancement in Medical Instrumentation level III barrier protection were introduced into the operating room workflow in 3-week phases; these gowns were lighter and more impervious than traditional cotton gowns. Each pilot was evaluated using a plan-do-study-act (PDSA) cycle. Primary end points were reusable gown use among sterile staff, cost, waste burden, and end-user satisfaction. End points were ascertained using audits, observation, and surveys. Reusable gown use was defined as wearing a reusable gown at least once. Cost comparisons included the purchase, sterilization, and waste disposal of each product. Barriers to reusable gown use were identified during the first PDSA cycle and addressed in the second PDSA cycle.

To estimate the cost and waste burden averted over 3 months and a year, operative logs were reviewed for all surgical facilities within the hospital system from January 2021 to June 2023. Surgical practitioners and scrub technicians who scrubbed into a case were included. Analysis was performed on Stata version 16.1 (StataCorp) in July 2023.

## Results

Two PDSA cycles involved 223 sterile staff (103 surgeons and 120 scrub technicians) covering 618 operations across 2 sites. Staff use of reusable gowns improved from a baseline of 0% to 88.6% (97 of 110) to 93.6% (110 of 118) over the PDSA cycles. Together, the 2 pilots reduced 371 pounds of solid waste and saved 2 cents per reusable gown (according to purchase prices quoted for the study site). End users who were completely satisfied increased from 22.9% (8 of 35) to 58.3% (35 of 60). Enthusiasm for broad adoption was especially strong among surgeons, although some declined to participate due to wary perceptions of sterility for cases involving implants, transplants, or chemotherapy infusion.

Barriers to reusable gown use included (1) workflow gaps in gown processing, (2) quality concerns regarding reusable gowns, and (3) uncertainty surrounding reusable textiles’ environmental impact. Addressing barriers included identifying nursing champions, educating prepilot staff on the gowns’ functionality and sterility, and mobilizing institutional leadership support.

When projecting reusable gown use for the hospital system per quarter, a mean (SD) of 10 491 (531) cases were performed with 37 690 (1871) operative staff, which could reduce solid waste production by 7538 (374) pounds and save $681 ($34) if reusable gowns were used (Table 1). Annually, a mean (SD) of 42 549 (2537) cases were performed involving 152 851 (9157) operative staff, which could reduce solid waste by 30 570 (1831) pounds and save $2762 ($165) by using reusable gowns. Cost comparison per gown is showcased in Table 2.

**Table 1.** Projected Number of Gowns Used Quarterly and Annually, With Corresponding Solid Waste Averted and Costs of Disposal Saved^a^

Metric	Mean (SD)		
	All sterile staff	Surgeon	Scrub technician
**Quarterly**			
No. of gowns	37 690 (1871)	22 432 (970)	15 257 (936)
Waste averted, lb	7538 (374)	4486 (194)	3051 (187)
Cost saved, $	681 (34)	405 (18)	276 (17)
**Annually**			
No. of gowns	15 2851 (9157)	90 744 (4419)	62 106 (4747)
Waste averted, lb	30 570 (1831)	18 149 (884)	12 421 (949)
Cost saved, $	2762 (165)	1640 (80)	1122 (86)

^a^ Projected numbers based on calculations for a tertiary hospital system in California.

**Table 2.** Cost Comparison of the Purchase and Handling of Reusable vs Disposable Surgical Gowns^a^

Item or service	Reusable gown cost, $	Disposable gown cost, $	Cost difference, $
Base product (per gown)	27.88	2.40	25.48
Waste disposal (per gown)	0.14	0.06	0.08
Sterilization (per use)	2.07	0.00	2.07
No. of cycles	75	1	NA
Total cost per use	2.44	2.46	−0.02

Abbreviation: NA, not applicable.

^a^ Cost values in table are informed by purchase prices quoted for the study site.

## Discussion

The US medical system is extremely wasteful and costly, harming the environment and human health.^5^ Using reusable surgical gowns could reduce the environmental impact in the perioperative space. If implemented, our projections suggest more than 30 000 pounds of solid waste would be averted annually by a tertiary medical center, with concomitant cost savings from landfill aversion. Limitations of this study include the single-center scope and the speculative nature of gown projections, which rely on sterile staff documentation in the medical record. Successful transition from disposable to reusable surgical gowns requires multidisciplinary workflow integration, staff education, and end-user investment, who at baseline may be ambivalent toward using reusable gowns.^6^

## Notes

Supplement.
Data Sharing Statement
