# Supplementary material for: Transition to Reusable Surgical Gowns at a Hospital System
Source: JAMA Netw Open. 2023 Aug 22;6(8):e2330246. doi: 10.1001/jamanetworkopen.2023.30246 (PMC10445178; doi:10.1001/jamanetworkopen.2023.30246)
Supplement: Supplement. — Data Sharing Statement [file jamanetwopen-e2330246-s001.pdf]

# Data Sharing Statement

Yap. Transition to Reusable Surgical Gowns at a Hospital System. *JAMA Netw Open*. Published August 22, 2023. doi:10.1001/jamanetworkopen.2023.30246

**Data**
**Data available:** No
